# An examination of social relations and concussion management via the blue card

**DOI:** 10.3389/fspor.2024.1392809

**Published:** 2024-06-03

**Authors:** Michael P. Jorgensen, Parissa Safai, Lynda Mainwaring

**Affiliations:** ^1^ Faculty of Kinesiology and Physical Education, University of Toronto, Toronto, ON, Canada; ^2^ Faculty of Health, York University, Toronto, ON, Canada

**Keywords:** sport-related concussion, participant welfare, match officials, culture of risk, rugby, sport policy, blue card, concussion recognition

## Abstract

**Introduction:**

Initially developed by New Zealand Rugby in 2014, the Blue Card initiative in rugby enables match officials to remove athletes from play if they are suspected to have sustained a concussion. Considerable attention has been paid by sport and health advocates to the possibilities and limitations of this initiative in safeguarding athlete health. However, little if any attention has been paid to the well-being of those responsible for administering the Blue Card (i.e., match officials). The aim of this paper was to examine match officials' experiences with and perspectives on implementing the Blue Card initiative in Ontario, Canada, with focused attention on the tensions around their ability to manage games and participants (e.g., athletes, coaches) while attempting to safeguard athlete well-being.

**Methods:**

Using Relational Coordination Theory (RCT) as a guiding framework and qualitative research method, we highlight the rich accounts of 19 match officials' perspectives and experiences regarding sport-related concussion (SRC) management and the Blue Card protocol.

**Results:**

Four themes were derived from the data, reflecting latent assumptions embedded within the concussion management process, which include: *assumptions of trust, respect, and cooperation*; *assumptions of shared responsibility*; *assumptions of shared understanding*; and *assumptions of harassment-free sport*.

**Discussion:**

Our findings emphasize the need to attend to social relations in concussion management and provide insight into match officials' fraught experiences on the frontlines of concussion management. We identify factors affecting match official well-being and provide considerations for concussion management initiatives designed to improve athlete safety, such as the Blue Card.

## 1 Introduction

The issue of concussions in rugby has, in recent months, come back to the forefront of public attention as media headlines track the evolving class action lawsuit against World Rugby, the international governing body for the sport of rugby union, filed by former players in the UK. Over 250 athletes suffering from neurological damage are involved in the litigation against World Rugby, as well as several national rugby bodies in the UK, seeking damages for what they argue is negligence due to a failure to protect (1). Though their specific symptoms and diagnoses vary, these athletes are united in their belief that their conditions are tied to head injuries sustained from their participation in rugby. Unlike similar litigation in the US against the National Football League (NFL), where plaintiffs argued the NFL intentionally misled the players on the dangers associated with football participation (2), this current case against World Rugby hinges on the argument that those in positions of authority did not do enough to mitigate these dangers in rugby (3). The lawsuit highlights the complex issue of duty of care in rugby and, at its core, a misalignment (real or perceived) of expectations of and responsibilities for protecting players' health and well-being.

Much can be unpacked about this specific lawsuit; however, we feel it is critical to highlight that this high-profile legal case magnifies the importance of understanding concussion management as a site of complex social relations and power dynamics (4) and not just as a medical issue in need of more clinical guidelines (5). Numerous scholars studying sport's “culture of risk” note that the social relations and interactions that form and are formed by sport shape the very attitudes, perceptions, and actions of the community as it relates to pain/injury tolerance [see (6, 7)]. For example, with regards to sports medicine clinicians, Malcolm [(8), p. 206] suggests that: “…not only clinicians’ behavior, but also their *understanding* of medical conditions, is shaped by the interdependent relationships in which they are enmeshed. Clinicians…come to internalize a definition of concussion that is similar to the way players and coaches understand the condition” (emphasis in original). Other scholars also point to the ways in which interdependent social relations between participants (e.g., athletes, coaches, clinicians, and referees) within sport communities facilitate or impede the management of sport-related concussions (SRC) (9–12). And yet, it is still very commonplace to see the issue of SRC narrowly framed as a medicalized and individualized health problem [(13); see also (14)], and concussion management fixated on prescribing and proscribing specific athlete behaviours to reduce risk (15).

We do not dispute the significance of concussions within rugby given the inherent nature of the high-impact contact and collision sport (16). The concussion rate among both male and female high school rugby players is high and often the result of tackle events during competition (17–21). At the community level, research found that 37.2% of a sample of Canadian senior rugby players reported experiencing concussion symptoms over the past season, 87% of which were formally diagnosed with a concussion (22). Additionally, rugby players are believed to be at an increased risk for neurodegenerative diseases relative to the general population [(23); see also (24)]. Rather, we aim to draw attention to the limitations of concussion management initiatives that do not adequately take the social relations and power dynamics between sport participants into account. We particularly highlight the lack of attention paid to the experiences and perspectives of match officials given how, in some jurisdictions, they are increasingly being called upon to take on prominent concussion management roles through initiatives like the Blue Card, while simultaneously playing an intermediary role in the very social context that makes such management difficult, or dangerous.

The Blue Card initiative in rugby enables match officials to remove players from play if they are suspected to have sustained a concussion. In Canada, the Blue Card initiative was introduced in 2019 by Rugby Canada amidst mounting public concern following the death of Rowan Stringer. Rowan was a Canadian high school rugby player who acquired multiple concussions in the same week without informing her parents or coaches and passed because of Second Impact Syndrome (25). In efforts to protect the welfare of community rugby athletes, Rugby Canada introduced the Blue Card process to all levels of amateur rugby competition in Ontario in the 2022 club season (26). A match official issues a Blue Card to an athlete when they suspect that athlete may have sustained a concussion, thereby removing them from play and initiating a return-to-play process preventing the athlete from returning to competition without medical clearance.

On one hand, introducing this process to facilitate the removal of an athlete from competition due to a suspected concussion is an important step towards safeguarding athlete health and well-being. On the other hand, the Blue Card initiative presumes a high degree of cooperation among key actors (players, coaches, and match officials) during games and a shared understanding that the safeguarding of athletes' health is *always* the common goal among *all key actors* at *all times* (27). Such a presumption is troubling when we recognize that the expanded responsibilities for safeguarding athletes' health in the Blue Card initiative are focused on just one group of actors involved in managing concussions (i.e., match officials). Research on the implementation of the Blue Card in other rugby nations (i.e., New Zealand) found that referees felt prepared to take on the added responsibility of the Blue Card and that it would not influence their enjoyment in their role (28). However, there remains a pressing need to better understand the relational dynamics involved in concussion management from the perspective of those responsible for its administration (i.e., match officials) and within the Canadian sport context. Any mechanism that relies on the decision of a single actor elevates the individuals in that role while neglecting both the social relations and power dynamics that underpin SRC and the implications of such an initiative on the health and well-being of the match officials themselves.

Considerable attention has been paid by sport and health advocates to the possibilities and limitations of the Blue Card initiative in safeguarding athlete health (28, 29). However, a paucity of attention has been paid to the well-being of those responsible for administering the Blue Card (i.e., match officials) amid a competitive sport context and culture that is replete with antagonism towards—if not outright hostility and harassment/abuse of—referees. This paper highlights findings from a study examining the relational dynamics within concussion management in Canadian community rugby through the experiences and perspectives of match officials, an underrepresented group in the sport research literature (30). In doing so, our work addresses a need for research evaluating concussion policy implementation (31) and match official well-being (32). Using Relational Coordination Theory (RCT) (33) as a guiding framework and semi-structured interviews, we examined the rich accounts of 19 match officials' perspectives and experiences regarding SRC management and the Blue Card protocol in community rugby.

## 2 Method

### 2.1 Theoretical approach

This study was conducted as a component of a larger, ongoing project exploring the implementation of the Blue Card protocol in Canadian community (amateur) rugby. Situated within a constructivist paradigm, this specific study focused on match officials' experiences with and perspectives on implementing the Blue Card process. A constructivist paradigm assumes ontological relativism, recognizing the presence of multiple realities and claims to truth, each constructed within the individual's mind through subjective interpretations of personal experiences (34). Transactional and subjective epistemological assumptions emphasize the social construction of knowledge throughout the research process (35). The researchers and participants engage in the research and co-create knowledge and meaning through double hermeneutical practices (36).

Authors MJ and PS have backgrounds within the Canadian rugby community (e.g., athlete, coach, match official). Author LM is a clinical psychologist with extensive experience working with athletes, specifically in the context of SRC. All authors have a background in research in health and sport injury. In adopting a constructivist paradigm, we acknowledge how our own beliefs and assumptions as researchers can influence the research process from the initial conceptualization of the study to the interpretation and subsequent presentation of participant experiences.

With foundations in social psychology, RCT is well-aligned with the paradigmatic and methodological approaches adopted in this study and offers a framework for understanding the relational dynamics involved in the concussion management process. The theory has been applied across various contexts, including sports, public health, and education (37). Gittell (38) defines “relational coordination” as “a mutually reinforcing process of interaction between communication and relationships carried out for task integration” (p. 301). Put differently, a social and relational process that informs and is informed by those involved—a reciprocal influence akin to the shared construction of knowledge through experience and social discourse advanced by a constructivist approach. According to RCT, the quality of relationships between participants, their respective roles in the process, and quality of communications influences their ability to coordinate action effectively (33, 39).

### 2.2 Recruitment

Following approval from the University of Toronto Health Sciences Research Ethics Board (protocol #39402) in September 2020, purposive sampling was used to select and recruit participants. Nineteen participants were recruited through the personal network of the first author and with the support of Rugby Ontario and Rugby Canada. Rugby Ontario facilitated recruitment by emailing all 42 match officials on their 2020 mailing list in September and all 223 match officials on their 2021 mailing list in March, April, and May. Inclusion criteria included registration as a match official in 2019, being 18 years of age or older, and having direct experience with, or knowledge of, the Blue Card during the 2019 season (the 2020 season was canceled due to the COVID-19 pandemic). Participants who met the inclusion criteria expressed interest by emailing author MJ. Upon obtaining consent, the researcher presented a brief demographic survey to the participant for completion and scheduled the online interview. The demographic survey included nine questions regarding the participants' age, gender, years of experience as a match official, current level of certification, most recent recertification year, level of rugby most qualified to officiate, years involved in rugby, personal concussion history, and other roles within the rugby community (e.g., parent, athlete, club official, etc.).

### 2.3 Data collection

Semi-structured interviews facilitated in-depth personal accounts of match officials' experiences with and perspectives on the Blue Card initiative. Interviews, lasting between approximately 50–80 min, were conducted virtually on the Microsoft Teams video communication platform, and recorded using an external Sony ICD-UX570 digital voice recorder. Interviews were conducted by author MJ, who adopted the role of a “passionate participant” in the research process in line with our constructivist approach (36). The interview questions were piloted in a mock interview with an independent researcher to verify the accuracy, relevance, and orientation of the interview guide to our research aims. The independent researcher was a behavioural psychologist and registered rugby match official. After completing an initial round of interviews (*n *= 6), we refined the guide to account for topics that arose organically during the interviews but were previously unaccounted for in our questions. We then conducted member reflections via follow-up interviews with most (*n* = 5) of the participants initially interviewed, allowing them the opportunity to clarify and expand on the topics discussed in the initial interviews.

The final interview guide (Supplementary Material S1) included rapport-building questions as well as orienting questions designed to ensure that those who hold or have held multiple roles within rugby were reflecting chiefly on their experiences as a match official when responding to interview questions (e.g., “Could you share an experience in that role as a match official that stands out to you?”). Four key areas were probed regarding participants' experiences and perspectives concerning SRC management and the Blue Card: (1) participants' understanding of their role in the SRC management (e.g., “What expectations are placed on match officials regarding concussion management?”); (2) participants' experiences and perspectives regarding the actions of rugby community members and their influence on the SRC management process (e.g., “How have athletes and other sport community members reacted when an athlete is removed from play due to a suspected concussion?”); (3) participants' beliefs about the responsibility for player safety among members of the rugby community (e.g., “Who do you think is most responsible for ensuring the safety of the athletes?”); and (4) participants' SRC knowledge (e.g., “Please describe your understanding of sport-related concussion injuries”). Follow-up questions and prompts were used to encourage the participants to expand on their anecdotes and accounts.

### 2.4 Data analysis

Following verbatim transcription, the data were analyzed according to the guidelines for reflexive thematic analysis as outlined by Braun and Clarke (40). Reflexive thematic analysis embraces the role of the researcher within the analytical process, recognizes researcher subjectivity as an analytic resource, and is well aligned with a constructivist approach (41). The researchers used the RCT as a sensitizing framework following the initial coding phase. Doing so provided an opportunity to capture details about the participants’ accounts that the theory may not adequately capture. We did not seek to validate the features of RCT.

Following the guidelines proposed by Braun and Clarke (40), the authors MJ and PS familiarized themselves with the data through ongoing critical engagement. Author MJ began this process during the transcription phase. Transcripts were shared with author PS and both authors engaged in independent, organic data readings. Discussions between authors MJ and PS began when each researcher felt sufficiently familiar with the data.

The analysis then shifted to the initial coding of the data, which resulted in specific codes such as *trust*, *role*, *intervention*, *pressure*, *knowledge*, and *respect*. These codes were developed through ongoing discussions between authors MJ and PS. RCT was used by the researchers as a sensitizing framework for examining the social and relational qualities of task coordination. Specifically, initial inductive codes were then compared to theoretical concepts described by RCT (e.g., shared goals, shared knowledge, and mutual respect). The researchers did not attempt to match the codes to the RCT concepts deductively, but used the overlap as evidence that they were giving sufficient consideration to the social and relational qualities involved in the coordination of a task. Throughout this process, author LM served as a critical third-party to challenge and suggest alternative interpretations of the data. In the next phases, codes were organized into themes which were then reviewed, refined, defined, and labelled. Finally, the authors organized and expanded the analytical commentary alongside select quotes to produce the final written work.

In efforts to ensure rigor, we adopted a relativist approach which suggests that researchers should critically curate the most relevant criteria to their study (42, 43). Braun et al. (41) provide a list of criteria to judge the quality of their specific approach to (reflexive) thematic analysis. We have adopted the list proposed by Braun et al. (41) to ensure coherence between the method and criteria for qualitative rigor. For example, we ensured that each data item received equal attention, that the themes were not developed from a few instances but were comprehensive and inclusive, and that we identified all possible extracts for our themes. Braun et al. (41) suggest that the data must be interpreted rather than paraphrased. Our interpretations led us to explore the underlying assumptions implicated in the Blue Card and concussion management more broadly, which were brought together alongside illustrative extracts in the following section to present a convincing story about the data and topic.

## 3 Results and discussion

The nineteen participants represent approximately ten percent of all match officials contracted by Rugby Ontario in a given season. Twenty-four interviews were conducted; five participants completed initial and follow-up interviews. The majority of participants identified as male (*n *= 14). Two participants identified as female, and one identified as non-binary. Two participants did not provide demographic information. Participants' ages ranged from 20 to 57 years (*M *= 39) and most (*n *= 15) reported having personal experience with a concussion injury. Participants' experience as a match official ranged from two to 23 years. The level of competition participants felt confident officiating ranged from junior to international, and the level of certification ranged from 1 (beginner) to 3 (experienced). Participants also reported having additional rugby roles (e.g., athletes, parents, coaches, administrators, and volunteers). Participants were required to complete SRC training through online modules provided by World Rugby as part of the match official certification process. Pseudonyms are employed in this paper to maintain confidentiality.

Our analysis highlighted not only the need to pay better attention to the social relations of concussion management, but to the negative consequences borne by match officials tasked with administering a concussion management program that is loaded with assumptions about risk tolerance and responsibility. Our findings suggest that, despite good programmatic intentions, concussion management efforts that do not attend to social relations and power dynamics in sport are bound to be insufficient so long as concussion is viewed as a solely clinical issue and so long as responsibility for concussion management is downloaded to but one group of actors. Specifically, the Blue Card downloads responsibility for concussion identification onto match officials; the administration of a Blue Card to remove an athlete with a suspected concussion sets into motion the subsequent concussion management pieces (e.g., medical clearance) within the initiative. Furthermore, as our participants shared, when considered alongside sport's culture of risk and pre-existing issues of harassment or abuse of match officials, the concussion management initiative's insensitivity to social relations in sport has troubling implications for the welfare of match officials themselves.

Our analysis developed four themes reflecting latent assumptions embedded within the concussion management process in community rugby from the perspective of match officials. The four themes include: *assumptions of trust, respect, and cooperation*; *assumptions of shared responsibility*; *assumptions of shared understanding*; and *assumptions of harassment-free sport.* Each theme was framed around participant accounts of relational disruptions within concussion management that challenge and, in some cases, contradict the logic of these assumptions. Select quotes from participant accounts were chosen to illustrate and substantiate the four themes; quotes have been mildly edited to assist with ease of reading.

### 3.1 Assumptions of trust, respect, and cooperation

Concussion management initiatives like the Blue Card presume those involved will work together for the benefit of the athlete (44). Study participants indicated that concussion management initiatives like the Blue Card assume, and in fact demand, a high degree of trust, respect, and cooperation among those responsible for implementation. However, participants' accounts suggested varying degrees of skepticism and suspicion around other actors' (e.g., coaches, athletes, medical or paramedical clinicians) intentions and actions during matches. In fact, our study participants' experiences suggest trust, respect, and cooperation are the exception rather than the norm or are extremely dependent on pre-existing relations and rapport with athletes and coaches:

Oftentimes, it's dependent upon your rapport with the players. So, in a lot of cases, in situations where I've shown up and people don't know who I am or know what my refereeing style is, like, “Who the hell is this person? Why does she think she can referee this game?” And then oftentimes when you return and they have trust in you and you've built that trust they're like, “Okay, she knows what she’s talking about,” and then oftentimes you get the desired response without even having to say it. (Phoebe)

Match officials indicated a sense of hesitancy to fully rely on and trust others whose cooperation in the Blue Card process was integral. Dallas expressed concern regarding the assumptions of trust and cooperation inherent in the return-to-play stage of the Blue Card process:

Whoever is doing the testing is evaluating that player and then deciding whether they can move on to the next stage in the rehabilitation process, which seems to be really safe. But you're still relying on somebody to do that. So, you're placing your trust in someone having that players' welfare in mind, rather than getting them back to the pitch too soon.

Participants recognized the value of positive, trusting, and cooperative relationships with others (e.g., coaches) to support concussion management. However, when reflecting on their on-the-field experiences in administering the Blue Card protocol, study participants spoke to ways in which trust gets eroded in concussion management because of their constant need to question other peoples' intent. Terry offered: “If you see someone stumbling around and clearly not all there, it becomes a responsibility to take action, even if the coach doesn't want to take that player off the field because they’re the star player.” Blake described how he has felt the need to question the accuracy of communication from others, which can have implications for trustworthiness (33):

You're always going to get people, coaches, assistant coaches, and players, they can say, “Hey man, that’s not true. This didn’t happen. I [saw] this happen. It didn’t happen like that.” Yeah, at the end of the day what's in your head, what you sell, whether it's wrong or whether it's right, you have to own it. The safety of the player will always steer you in the right direction at the end of the day. (Blake)

In addition to the need to constantly question players and coaches during matches and the impact of that on their sense of trust and cooperation, participants also often spoke to their need to protect themselves from criticism and to defend their decisions in the context of removing an athlete from play due to a suspected concussion; this also counters, if not erodes, assumptions of trust, respect, and cooperation underpinning the Blue Card initiative. When discussing the process of administering a Blue Card, Casey explained how match officials often need to “reduce the amount of potential backlash from the clubs that comes at a critical moment.” Yasmin echoed this sentiment:

There's worry because there's a lot more backlash. This is taking a player out for a prolonged period of time and that increases risk of emotion coming out and players and coaches and it definitely causes stress. I don't know if other people feel that way but I definitely do. It definitely scares me to have to use my cards. I just don't want to deal with the anger from the players or whatever may come of it.

Study participants routinely commented on how match officials can be at odds with coaches and athletes when deciding to remove a player with a suspected head injury making plain that, while trust, respect, and cooperation between players, coaches and match officials are critical to the success of concussion management initiatives, these are not always present. Our findings underscore the relational nature of trust (45) and its dependence on relationships between individuals within systems for the purpose of task coordination. Our study participants themselves noted the importance of relationships in concussion management, “I think it would be more to do with developing that relationship to have [a discussion regarding the removal of an athlete from play due to a suspected concussion], you know, if and when that needs to happen” (Riley).

Gittell (33) argues that relational coordination can yield the most significant returns in contexts where conditions of reciprocal interdependence and uncertainty are present and when there is a premium placed on the responsiveness of a process (i.e., urgency). Participant accounts suggest that uncertainty and urgency exist within the concussion identification process due to the internalized injury experience, potential lack of physical signs, and the athletes' desire to play through injury or prematurely return to competition.

However, our participants' accounts highlighted the very ways in which the game-time dynamics and context worked against the building of positives relations. For example, we cannot disregard that the Blue Card expands the tools possessed by match officials—it affords them more power and capacity to intervene in the game. It is not unreasonable to appreciate that other sport participants may become hostile to the expansion and use of these tools, viewing them as a “punishment” (Louis) and an even greater imbalance of power. Study participants noted that this (mis)perception impacts their interactions with athletes; as Lukas explains:

It's not a Red Card. You aren't getting sent off because it was a dirty or illegal play. You're getting sent off because you took a big hit. These guys are fuckin' builders and plumbers. These guys are hard, big men; they get aggressive, “What? There’s nothing wrong with me!” I can see it not going well.

Furthermore, community rugby match officials are often the sole referees on the field. Multiple demands fed into study participants' feelings of urgency and uncertainty when managing concussions and the game more broadly. For example, Jordan noted the challenge of balancing one's attention to a potential injured athlete and ensuring the safety of those who continue to compete, “It's very difficult to assess signs and symptoms when the ball has already moved 25 meters away. You’re looking at that contest on the ruck for potential foul play.” When an athlete is injured in rugby, match officials often allow play to continue until a natural stoppage occurs, if there is no further risk to the injured athlete. We speculate that these power imbalances and logistical demands hinder the development of trusting, respectful, and cooperative relationships.

### 3.2 Assumptions of shared responsibility

Our analysis of study participants' accounts highlighted tensions around the notion of shared responsibility. On one hand, participants were cognizant of their responsibility as match officials to ensure the safety of athletes during competition; Dallas noted: “You’re meant to be the person in charge. If you don't feel comfortable with it, don't be an official, but that's your job to make sure that the players are safe.” On the other hand, participants indicated that responsibility for concussion management must be shared: “[Concussion management] can't just be placed on the referee, it's the community itself that needs to be responsible for it” (Eddie). However, participants were frustrated with other key sport actors for failing to accept or act on their responsibility for concussion management decisions in their respective roles (e.g., coach, therapist, captain, athlete). As Blair shared:

I think the referee on the day is the person who makes the final decision. So, they have to be the most responsible. Inevitably, if that person, the coach, or the player, or the captain, or the [trainer], doesn't want to make the decision, then it's up to the referee.

The participants described amplified demands on match officials when they were obligated to act as *last-in-line* decision-makers when those in adjacent roles (e.g., coaches, medical staff) did not.

Participants viewed the formalization of the match official's role in concussion management (e.g., the implementation of the Blue Card) as a direct result of others' inaction; this finding itself closely related to faulty assumptions around trust, respect, and cooperation noted above. Although Eddie was willing to take on this extra burden, he was quick to lay blame at other actors' feet: “What we’re managing here is unfair practices by team management, right? Really, that's what we’re doing and I’m actually okay with that.” Other study participants, however, were far more worried about the potential consequences of growing demands (e.g., blame, liability, recruitment, and retention):

I gotta make the decision at the end of every day. If a kid comes back and then it's like something drastically happened, who are they gonna blame? The referee. “Why would the referee not stop the game? Why did the referee not send them out?” They won't look at it and go, “Well, there was an athletic therapist there.” No, they'll be like, “What was the referee doing? That’s his job.” (Jackie).

For some participants, the tensions around assumptions of shared responsibility arose out of concerns regarding their perceived lack of qualifications to identify suspected concussions as compared to other actors (i.e., medical/paramedical clinicians); more than half of participants noted that they were “not doctors” at some point in their interviews. This contrasts Sullivan et al. (28), who reported that most of the match officials in their sample felt prepared to take on the additional responsibility of the Blue Card. While Rugby Canada did provide some training and education on the Blue Card, these were often presented solely as online information sessions or modules. Several participants noted that on-field development sessions for match officials rarely included the opportunity to go through the process of issuing a Blue Card and what this might look like when other participant groups are involved (e.g., coaches).

While several participants reasoned that those with greater medical knowledge should be more prominent in concussion management decisions, others continued to express skepticism or suspicion: some teams have “a really good [therapist] that does their due diligence or there's gaps, right? So, I’ve seen both to be perfectly honest.” (Jordan). Nevertheless, when others fail to act, the *burden of responsibility* ultimately falls on match officials regardless of their confidence in their expertise:

You have to take note of all this information. So, there's just so much going on in your head and, generally speaking, at the lower levels it's newer referees. So, these people are already overwhelmed and you're just adding another thing to their role. Now tell us when these people are injured, like when they have a brain injury. But we're not going to tell you how, we're not going to prepare you for that, but like, “Here you go, take this Blue Card and go do your job.” (Phoebe)

Participants routinely felt that they were left alone to act in situations where they did not hold the necessary or expert knowledge; and yet, were expected to hold ultimate responsibility for safeguarding athletes' health. Shared knowledge also facilitates relational coordination (33). Exclusive knowledge was perceived to exist between roles whereby experts in one functional area may develop exclusive knowledge through differences in training and socialization (e.g., concussion knowledge among medical support staff). Exclusive knowledge can cause division among those who hold distinct roles, further exacerbated by a culture of disrespect [e.g., match official abuse (46, 47)].

Identifying signs and symptoms of concussion can be challenging for those involved, and the uncertainty (or “grey area”) in this process was a steady source of stress for match officials. Objective signs (e.g., loss of consciousness) are not always present when a SRC has occurred, and symptom onset is typically delayed (48, 49). Furthermore, reliance on athlete self-reporting is problematic, as research suggests some athletes hold negative attitudes toward self-reporting concussion injuries (10, 50–54). These inherent challenges also informed participants' attitudes toward other actors:

It's tough because the match official is responsible for so much, but I know so many players try to hide things just to continue playing that it makes it difficult to put as much of that pressure on when they're actively working against us in that sense, you know? Concussions are also not black and white, it's a pretty big grey area. You're always going to be put in a bit of an awkward situation where you know that you're not the right person to be making this decision, but ultimately you are the person who has to make the decision. At the end of the day, I know that I'm not a doctor, but I also know that I don't want to take the risk on somebody's health too. I don't want to blow it. (Louis)

Louis questioned whether the decision to act should have fallen to match officials in the first place. While participants expressed concern for concussion identification responsibilities in the management process being downloaded onto match officials, responses such as Louis’ suggest match officials also attempt to shift responsibility onto others. Athletes were viewed as failing to accept responsibility for their well-being [cf., (9)], while other actors were viewed as being the “right” people to make concussion management decisions but who often failed to do so. Related research by Zanin et al. (12) identified specific strategies (*Agentic Denial*) used by those involved in concussion management to obscure one's agency in concussion events—diminishing the responsibility and culpability of the actor. These concerns highlighted by our participants and other research (12) emphasized the social and relational consequences of downloading responsibility in concussion management onto others. As Cameron stated:

I felt like at that point, that player was at risk for injury, ‘cause I felt that she at least had a mild concussion or at least enough to warrant being sent off the pitch. And the last thing you want, ‘cause this is after Rowan’s Law, you know, which was huge with all the different [professional development] on it, I think there are still some people that weren’t quite on board with it all, or at least the severity of it. And the last thing you want, like I was feeling responsible for the fact that athlete was still on the pitch, even though I had no control over whether she got the follow up or what. There’s too many things out of my control, but I still felt responsible that she was on field and I didn’t feel it was the right decision.

In sum, match officials believed that a shared sense of responsibility was not fostered among participant groups—contradicting embedded assumptions and disrupting the relationships of those involved in the concussion management process (e.g., removing an athlete with a suspected concussion). Rather, downloading the final decision to the match official has provided the opportunity for others (e.g., athletes and coaches) to avoid responsibility. Salmon et al. (55) have similarly identified responsibility concerns in coordinating concussion management. Match officials accepted their role in ensuring the safety of participants, aligning with Clacy et al. (56). However, they felt the *burden of responsibility* through others' inaction (i.e., letting the match official make the final decision) or active resistance (i.e., hiding concussion symptoms). In conjunction with these attitudes was the concern that match officials would be the ones to endure the consequences should something catastrophic occur (e.g., the Rowan Stringer incident), as illustrated by Cameron.

### 3.3 Assumptions of shared understanding

In sharing their experience with implementing the Blue Card protocol in games, match officials' accounts pointed to an inherent assumption underpinning concussion management—that athlete welfare is prioritized above *any other* objectives or goals, such as winning, by *all* key actors at *all* times. However, participants routinely shared concerns regarding others' negative perspectives of match officials. Specifically, match officials believed coaches and athletes viewed them as antagonistic to their personal or team goals (e.g., being able to compete, winning the competition). The oppositional framing of participant groups implies relational disruption among key decision-makers where “the only one that's ever happy if it happens [i.e., a match official removes an athlete due to a suspected concussion] is the opposing captain, right?” (Riley). Louis shared:

[Coaches are] kind of thinking, “Well, I don’t want to send that guy off, he’s really good. I don’t want to lose the game.” Because ultimately, their ultimate goal in the course of the game is to win. So, you have that issue where [coaches are] not going to take [concussion management] as seriously or they may not make the right decision for the individual because they want to make the right decision for the game. (Louis)

Regan's suspicions of athletes' motivations to resist match officials' efforts to safeguard their brain health are foregrounded when they note: “[Athletes] just want to go back in the game and just keep playing and ignore the head injury, right? You could ask them, but they’re not very reliable. They’re not very trustworthy because they just want to play the game.” Louis' and Regan's perspectives challenged the assumption of shared understanding among coaches and players whereby they did not believe that others would deprioritize performance-related goals in the event of a suspected concussion. Experiences of hostility from others, including the fear of backlash noted earlier, seemed to entrench these beliefs and disrupt concussion management efforts.

Our findings aligned with extant research [e.g., (10, 57)] describing a functional understanding of SRC among amateur and sub-elite rugby players who downplayed injury severity and focused on returning to competition in the short-term rather than recovery or long-term health. Given the ways in which health is equated to performance, whereby health becomes reduced to the ability to perform excellently [cf., (14)], participants' accounts suggested that athlete welfare was viewed as emotional welfare, connected to the desire to remain in competition, rather than brain welfare. Riley suggested that the issue of athletes prioritizing performance goals is embedded within the broader culture of sport:

Like at the end of the day, [in] the perfect world, the players are pulling themselves off and saying, “My head hit this tackler’s knee and it hurts. I’m not dizzy or anything like that right now, but I don't want to risk it.” The culture doesn't allow, allow is a strong word, encourage, the culture doesn't encourage it. This isn't a rugby comment, this is hockey, this is football, it doesn't encourage that, right?

Athletes' drive to play through injury is not unique to SRC or rugby. However, the normalization of pain and acceptance of playing injured is part of broader socio-behavioural norms inherent in sport whereby competitors are encouraged to push boundaries in the pursuit of performance (58–60) and, by extension, view those who enforce the rules that interfere with their tolerance of the culture of risk as antagonistic to their performance goals (61, 62).

Participants believed that athletes would view intervention via the Blue Card as a threat to their ability to compete and as an inadvertent benefit to the opposing team. Where they could, the participants reaffirmed their neutrality: “The match official doesn't care win, lose, or draw what the end result is” (Louis). However, even at the highest level of competition, rugby match officials have been found to display implicit bias in their decision-making (63). Terry argued that this could impact the process of onboarding athletes to new concussion management practices like the Blue Card:

I think it's a little harder to sell the players because the Blue Card is giving the referee a tool to send them off without complaint. In theory, we can do that right now, but it's a more formalized process that can keep them out several games, whether or not they recognize that it's their welfare that we're worried about. I think players are a little more concerned about how it's gonna influence their ability to play matches.

Participant accounts indicated tensions between athletes' personal autonomy (i.e., respect for the athlete as a decision-maker) and match officials' professional autonomy (i.e., respect for the match official as a decision-maker). Participants emphasized that they were “out here for the welfare of [athletes]” (Sonny). However, they also expressed concern that athletes may view match official intervention through the administration of a Blue Card as a form of paternalistic intervention—a threat to their agency. Cameron insisted that he will “use [the Blue Card] appropriately. I’m not gonna abuse use it.” Nonetheless, uncertainty is inherent to concussion management and is experienced by athletes (64) and all those who make decisions on their behalf (8, 65, 66).

Participants anticipated additional resistance in situations where their decision to remove an athlete due to a suspected concussion was not agreed upon by medical staff, further challenging assumptions of shared understanding. For example, Lukas explained: “If the [therapist] says, “No, this athlete is fine,” but you feel this player does not look good, there's going to be conflict.” Situations with conflicting opinions between match officials and medical staff may reaffirm athletes' perspectives of match officials as antagonistic to their goals. In contrast, bringing others into the decision-making process may help reposition concussion management efforts as collaborative rather than authoritative and ease hostility toward match officials:

…now players are thinking of you less as being hostile to their goals. I guess, like, okay, “That was a decision that was made by the captain. Well, that clearly means that it was the right call.” As opposed to, “Ah yeah, the ref just blew that call, and you threw out our best player.” You're not the antagonist in that situation. Everybody collectively made that decision. Then you don’t have that hostility flowing for the rest of the game. (Louis)

According to the RCT, shared goals contributes to the quality of the relationships among those involved in task coordination (33). When the goals of participants are congruent with one another, a bond is formed that enables them to more efficiently adjust to new information. However, goal discrepancy can exist between roles. In our sample, the functional (i.e., performance) goals of coaches conflicted with the process (i.e., professional) goals of the match official in events where an athlete was removed due to a suspected concussion (e.g., via the Blue Card process).

Match officials challenged the assumption of a shared understanding that *every* member of the rugby community *always* prioritizes safeguarding athletes' health. Key actors, such as captains, coaches, and teammates, have unique epistemic insight into an athlete's values, motivations, and goals. These social agents play a central role in athletes' negotiations of risk in sport, including their cooperation or resistance to the protective goals of the concussion managers. Without proper consideration of the social relational issues in concussion management, we contend that initiatives like the Blue Card will, at best, fail to safeguard athletes and, worse, inadvertently risk the welfare of others.

### 3.4 Assumptions of harassment-free sport

Participants expressed concern regarding acts of abuse endured by match officials by other actors (e.g., coaches, athletes), challenging the assumption that match officials can implement concussion management without harassment or worse. Although there is ample evidence in the media and extant scholarship documenting the ubiquity and pervasiveness of abuse directed toward rugby match officials across all levels of rugby (and other sport) competition (67–71), initiatives like the Blue Card pay no heed to this issue as a critically important consideration in concussion management. Rather, harassment and abuse are viewed and treated as separate issues from concussion management. The assumption that match officials can implement the Blue Card in harassment-free sport underscores the lack of attention to the social relations of concussion management and to the risks to match officials' own health and welfare.

Despite some participants being committed to the belief that rugby promotes respect towards match officials—“One of the nice things why I like rugby is the respect for the referee or at least respect for their decisions” (Blair)—others shared examples of having been personally subjected to abusive acts in their role as match officials, which were broadly described as salient and negative emotional experiences. Others expressed concern about the potential for increased abusive behaviour following the implementation of the Blue Card: “…you know what happens. Your team loses, “It was the Blue Card, [it was the] referee!” Verbal abuse, ya know? So, it can be tough” (Lukas). What was noteworthy for us was the degree to which study participants reframed experiences of abuse as things that needed to be tolerated and how they downplayed the severity of these events by describing them as a *normal* feature of their role and work [cf., (72)]. Our participants' accounts reflected this normalization of abuse toward match officials: “I found that as soon as you blow the last whistle, the coaches do relax a little bit more and the players too. They’ll be angry at you, but then that's life of a referee, right?” (Lukas).

Though abuse can come from a variety of actors (e.g., parents and spectators), participants identified coaches as common perpetrators of match official harassment and abuse [cf., (70)]. As Riley shared:

The coaches have huge role, right? There's the good ones and the not-so-good ones, and the personality of the coach can very easily be taken on by the team. In the examples where the not-good-ones are standing on the sideline, and when I say “not-good-ones”, they may be good coaches, Xs and Os, motivating, those type of things, but they are not good for the game, right? They are not good for delivering teams that play with respect, and they're not good at respecting themselves or showing respect themselves, and they're the ones who are yelling and screaming on the sidelines on every 50–50 call, and they are screaming the loudest when their own team makes a big hit.

Several participants reflected on how coaches serve as models of acceptable behaviour for athletes, and that the actions of coaches can perpetuate an environment where match official abuse is viewed as acceptable. Yasmin expanded on this, particularly in connection to her experience as a female match official:

Coaches should also be trying to display a positive relationship with referees because that also determines how players are going to treat referees in future or in that game specifically. It is really daunting for a lot of women to look at a situation and recognize that they may have to tell a big 50-year-old man that their star player can’t continue playing. And that's a fear that I have every time I ref a match. It's just like, “What if this coach just loses it on me?” And every time I'm leaving, I'm like, “I want to get out of here.”

Yasmin, one of two female match officials in our sample, was the youngest match official interviewed. Her experience directly aligned with other research on match official abuse documenting increased rates of harassment and abuse directed toward younger (73), less experienced (74), and female officials (75, 76), suggesting their experiences of abuse can differ in both type (e.g., gendered abuse) and frequency from their older, more experienced, and male peers, subsequently impacting their mental health and retention in sport.

The stress-inducing nature of administering the Blue Card, even just the thought of potentially having to administer it, was prevalent among participants given an expectation that their efforts and decisions would be met with hostility. What stood out to us was the ways in which a few participants attempted to manage the real or anticipated tensions, and cope with the stress that was induced, by adopting a more threatening posture with coaches and athletes. For example, Sonny, a male match official who was among the oldest and most experienced in our sample, shared what he communicates to coaches and athletes to make it clear that they must work with him in managing suspected concussion events and the game more broadly:

If we can't work together then it may not be a good day for you. It may not be a good day for your team if you choose not to help diffuse the situation. If you choose not to be active in making this game move in a certain way, then it could be harsher on your team, right? It could be more difficult on your team if I've got to do it myself, right?

Logan shared a similar positionality when he stated: “Coaches think they are big and sometimes you gotta kind of bring them a notch down.” In these excerpts, we see participants attempting to exert their role-given authority but doing so in, frankly, aggressive, *quid pro quo*-type language. We do not disregard how these verbal tactics are maladaptive, but recognize the impetus for this approach—the lack of attention in the concussion management process to the fraught power dynamics between sport actors and to existing incidence of harm of match officials, and the faulty assumption embedded into the Blue Card that match officials' attempts to safeguard athletes' welfare will be accepted by others without any objection, harassment, or abuse.

When the quality of relationships (via qualities such as mutual respect) and communication among participants is high, relational coordination occurs (33). However, abuse of match officials reflects a lack of mutual respect between participants and is a common phenomenon in the context of amateur rugby (69, 70). Concussion management assumes harassment-free sport; participant responses challenged the soundness of this assumption. Research exploring coaches’ perspectives on match official abuse described the humanizing effect that strong relationships between coaches and match officials had in deterring abusive behaviours (47). However, current concussion management initiatives presume such a relationship in their absence of attention to the social relations of SRC. When concussion management initiatives like the Blue Card fail to adequately consider the complex social relations and power dynamics inherent in the process, match officials are prompted to try whatever they can to navigate the process. This includes using strategies in their toolkit that can be maladaptive, leading to further erosion of social relations and impacting the well-being of those involved.

### 3.5 Pulling the threads together

As highlighted above, inattention to the social relations and power dynamics that weave in and throughout competitive sport render concussion management initiatives as potentially dangerous for sport participants like match officials. Our study participants' accounts in implementing the Blue Card initiative shed light on the need to interrogate both intended and unintended consequences of concussion management programs, and the constant need to think expansively with regards to protecting the health and welfare of *all* individuals operating within organized sport. Despite the potential benefits to athlete welfare, the welfare of those responsible for administering such things as Blue Cards *in situ* is risked as their decisions and actions are embedded within a sport culture that far too readily tolerates risk, pain, injury, and the harassment (and, at times, abuse) of match officials (46).

In highlighting latent assumptions of trust, respect, and cooperation, of shared responsibility, of shared understanding, and of harassment-free sport, our findings underscore how current approaches to concussion management do not adequately account for the relationships among those involved. In the Blue Card initiative, a great deal of responsibility is downloaded onto match officials and these actors are required to serve as the gatekeepers of the process. Yet, concussion management demands common understandings of concussions, shared commitment to health always, and cooperation among all sport actors (e.g., athletes, coaches, and medical/paramedical clinicians). The existing Blue Card initiative assumes these features are in place, but our study participants' experiences and perspectives challenged such assumptions. Our findings also contrast those of Sullivan et al. (28), who found that most of the match officials sampled felt prepared to take on the role of on-field concussion gatekeeper and did not think that this would affect their satisfaction with refereeing.

Examining our participants' experiences through the lens of RCT afforded us opportunity to appreciate the importance of high-quality communication and relationships among individuals as developed through shared goals, shared knowledge, and mutual respect (39). According to the RCT, task coordination is highest when participants' goals are congruent with one another such that a bond is formed that enables them to adjust to new information more efficiently (33). The successful implementation of the Blue Card initiative depends on reciprocal interdependence and multiple participants coordinating tasks across various stages of concussion management (i.e., identification, removal, recovery, return). When goal discrepancy exists between individuals, such as when the performance imperative of coaches and athletes conflicts with the concussion management responsibilities tasked to the match official because of the Blue Card protocol, task coordination falls apart. In addition, when there is a lack of mutual respect, for example, when in those instances of harassment or abuse directed towards match officials for simply doing their work, we see further deterioration of task coordination as well as an exacerbation of division and harm among sport participants. For example, our study participants candidly shared moments where they preemptively steeled themselves for the blame that they anticipated would be directed toward them by others. They shared reciprocation of blame by suggesting that others were at fault for the challenges they experienced regarding concussion management and, in some cases, adopted their own maladaptive coping strategies by being threatening.

When the relationships between key actors are nurtured, there is a humanizing effect on the perspectives of others (47). Fostering positive relations between participant groups may lower the risk of match official abuse (47) and support concussion management efforts (i.e., the Blue Card). Hancock et al. (77) challenges the assumption that officials inherently operate as a group due to the transient nature of group membership from game to game, and notes that familiarity heightens trust. In the context of the Blue Card, the match official depends on other participant groups to successfully facilitate the process. Therefore, we recommend integrated training and education involving multiple participant groups (e.g., match officials, coaches, athletes) should be developed to help facilitate relational coordination. Current approaches to training and education for the Blue Card and concussion management are primarily static and siloed.

Organizations have a duty of care to provide support through the adoption and active communication of a zero-tolerance policy (74, 78) alongside processes for reporting instances of match official abuse and follow-up support (46) to promote the retention of match officials in rugby. We recommend that sport organizations explicitly connect concerns for match official abuse to concussion management efforts. For example, concussion training and education initiatives should integrate discussions regarding match official abuse and outline the abuse reporting process alongside how to access the supports available for match officials (e.g., opportunities to shadow games and review film for instances of abuse). Future research could evaluate the effectiveness of such developments on outcomes such as the satisfaction and preparedness of match officials as examined by Sullivan et al. (28).

### 3.6 Strengths and limitations

It is important to note that, as data collection occurred during the COVID-19 pandemic, the number of match officials who registered during this time was substantially reduced (registration is typically greater than 400). Even though women account for approximately 18% of match officials registered with Rugby Ontario, our sample was not completely gender-representative of the broader match official population. Furthermore, the majority (84.2%) of our sample did not have experience administering or supporting a Blue Card administration. However, our sample may have accounted for all seven administrations of the Blue Card during the 2019 season. One participant personally issued four Blue Cards (57%) in our sample. While two other participants supported the administration of Blue Cards (three administrations and one administration, respectively) as assistant referees.

Though participants spoke briefly on the return-to-play aspect of the Blue Card and the concussion management process more broadly, their responses tended to focus on the coordination of the removal process as has been highlighted in this paper. This is not surprising as match officials are not involved in the return-to-play aspect of the concussion management pathway. However, it remains a noteworthy missing piece in the Blue Card concussion management pathway. The fact that match officials are not involved in the return-to-play stage of the Blue Card process but felt the need to share their lack of trust in others to manage these areas is an important finding.

It is essential that we acknowledge the potential for our insider experiences as members of the rugby community to shape our perspectives on this research topic and approaches to this study, specifically. For example, our insider experiences enabled us to better understand the specific language chosen by our participants to describe their experiences, including sport specific terms (e.g., scrum). To account for any potential limitations of our insider experiences (e.g., that we would not be sensitive to perspectives that differed from our own), author LM acted as a critical friend to challenge the interpretations of the data and encourage alternative viewpoints.

## 4 Conclusion

Our participants shared the ways in which they attempted to individually navigate a concussion management initiative that requires a much more collective and nuanced response. Part of this requires much greater critical attention to the implications of the still pervasive tolerance of a “culture of risk” alongside the continued dominance of the performance imperative in competitive sport, even at the community/amateur level. Part of this also requires careful examination of what we expect to have happen when such an initiative is siloed from other health and welfare issues, including match official harassment and abuse. Experiences of abuse impact the welfare and retention of match officials in sports (78, 79), as well as their ability to manage the match and ensure the safety of athletes (73, 80). Yes, we recognize that rugby governing bodies have responded to referee harassment and abuse by implementing strategies to safeguard match officials from the amateur level (81) up to international competitions (82). However, to date, the approach to concussion management as operationalized through the Blue Card initiative has occurred in a siloed manner disconnected from the attention being paid to match official harassment; we maintain that concussion management initiatives across all sporting contexts, not just rugby, must be critically evaluated in lock-step with other welfare programs to ensure that efforts to safeguard one group's welfare does not come at the expense of others' health and well-being.

## Data Availability

The raw data supporting the conclusions of this article will be made available by the authors, without undue reservation.
