# Supplementary material for: An examination of social relations and concussion management via the blue card
Source: Front Sports Act Living. 2024 Jun 3;6:1392809. doi: 10.3389/fspor.2024.1392809 (PMC11180777; doi:10.3389/fspor.2024.1392809)
Supplement: Supplementary file 1 [file Datasheet1.pdf]

Interview Questions

1. Introduction

Background:
Rugby is one of the most popular contact sport world-wide. Due to the substantial physical demands of the sport, there is a substantial risk of injury for those who participate. Managing these risks is a complicated process involving many stakeholders within the sport community.

Purpose:
The purpose of this research is to better understand the experiences of Match Officials regarding the management of injury risk in rugby, and more specifically the Blue Card process.

Outline:
This interview will cover questions relating to sport-related concussion injury, managing injury risk in rugby, the role and experience of Match Officials, and the Blue Card process. Each topic will include several questions and prompts.

2. Match Official: Orientation and rapport building

- □ To start, I would like to talk a little bit about your experience as a rugby Match Official and your interest in this study.
- □ So, what interested you in participating in this study?
- □ If you are comfortable, could you share an experience in that role as a match official that stands out to you?

3. Match Official Role: SRC management & the Blue Card

- □ What expectations are placed on referees in regards to managing injury risk? SRC?
- □ What signs would suggest a player should be issued a Blue Card and removed from play?
- □ Prompt: Envision yourself in a situation where you have to give an athlete a Blue Card.
- □ Outline your thought process when considering whether to issue a Blue Card or not.
- □ How do you anticipate the process going?
- □ Describe your level of comfort issuing a Blue Card.
- □ What other considerations might you have when deciding whether to issue a Blue Card?

- ◻ Now I would like to talk about the Blue Card. With these questions, I am hoping to better understand your thoughts and experiences on the process.
- ◻ Could you explain to me what the Blue Card process is
- ◻ Could you tell me about your experience with the Blue Card process?
- ◻ How do you view the Blue Card process in relation to the management of injury risk?
- ◻ What training was provided for you on the Blue Card process?
- ◻ Is there any additional training that you think would benefit Match Officials?
- ◻ Have you done any reading or personal research regarding the Blue Card process?

4. Actions of Others: Response to the Blue Card & SRC management

- ◻ How have athletes and other sport community members reacted when an athlete is removed from play due to a suspected concussion?
- ◻ Do you think athletes / others will follow to the Blue Card process?
- ◻ How might a referee deal with an athlete / other who react(s) negatively to the Blue Card / SRC management?
- ◻ What concerns do you have over the implementation of the Blue Card process?
- ◻ What impact do you think the Blue Card will have on rugby / SRC management?
- ◻ Do you have any experiences that you feel are unique to you as a female/male/non-binary Match Official?
- ◻ Compared to your female/male counterparts, how would you describe your experience managing injury risk in rugby?
- ◻ What factors contribute to your experiences?

5. Responsibilities: Managing risk / safety of participants

- ◻ As a Match Official, how do you view your responsibility in managing injury risk?
- ◻ How do you, as a Match Official, strike a balance between keeping players safe while ensuring you are not influencing the outcome of the game?
- ◻ How effective do you think referees can be in identifying high risk situations in games?

- ◻ Who do you think is most responsible for ensuring the safety of the game?
- ◻ How do you feel is responsibility shared among members of the rugby community?
- ◻ At what point is the match official no longer responsible for the safety of the players?
- ◻ What is the most important part of concussion management?

6. Concussion Knowledge

- ◻ I have a few questions regarding concussion injuries. With these questions, I am hoping to better understand your thoughts and experiences on them.
- ◻ Please describe your understanding of sport-related concussion injury.
- ◻ How do concussion injuries compare to other sport-related injuries?
- ◻ What are your thoughts on how concussion injuries are managed in rugby?
